# Coffee Leaf Rust Disease Detection and Implementation of an Edge Device for Pruning Infected Leaves via Deep Learning Algorithms

**DOI:** 10.3390/s24248018

**Published:** 2024-12-16

**Authors:** Raka Thoriq Araaf, Arkar Minn, Tofael Ahamed

**Affiliations:** 1 Graduate School of Science and Technology, University of Tsukuba, 1-1-1 Tennodai, Tsukuba 305-8577, Japan; 2 Institute of Life and Environmental Sciences, University of Tsukuba, 1-1-1 Tennodai, Tsukuba 305-8577, Japan

**Keywords:** coffee, production, pruning, deep learning, edge device

## Abstract

Global warming and extreme climate conditions caused by unsuitable temperature and humidity lead to coffee leaf rust (*Hemileia vastatrix*) diseases in coffee plantations. Coffee leaf rust is a severe problem that reduces productivity. Currently, pesticide spraying is considered the most effective solution for mitigating coffee leaf rust. However, the application of pesticide spray is still not efficient for most farmers worldwide. In these cases, pruning the most infected leaves with leaf rust at coffee plantations is important to help pesticide spraying to be more efficient by creating a more targeted, accessible treatment. Therefore, detecting coffee leaf rust is important to support the decision on pruning infected leaves. The dataset was acquired from a coffee farm in Majalengka Regency, Indonesia. Only images with clearly visible spots of coffee leaf rust were selected. Data collection was performed via two devices, a digital mirrorless camera and a phone camera, to diversify the dataset and test it with different datasets. The dataset, comprising a total of 2024 images, was divided into three sets with a ratio of 70% for training (1417 images), 20% for validation (405 images), and 10% for testing (202 images). Images with leaves infected by coffee leaf rust were labeled via LabelImg^®^ with the label “CLR”. All labeled images were used to train the YOLOv5 and YOLOv8 algorithms through the convolutional neural network (CNN). The trained model was tested with a test dataset, a digital mirrorless camera image dataset (100 images), a phone camera dataset (100 images), and real-time detection with a coffee leaf rust image dataset. After the model was trained, coffee leaf rust was detected in each frame. The mean average precision (mAP) and recall for the trained YOLOv5 model were 69% and 63.4%, respectively. For YOLOv8, the mAP and recall were approximately 70.2% and 65.9%, respectively. To evaluate the performance of the two trained models in detecting coffee leaf rust on trees, 202 original images were used for testing with the best-trained weight from each model. Compared to YOLOv5, YOLOv8 demonstrated superior accuracy in detecting coffee leaf rust. With a mAP of 73.2%, YOLOv8 outperformed YOLOv5, which achieved a mAP of 70.5%. An edge device was utilized to deploy real-time detection of CLR with the best-trained model. The detection was successfully executed with high confidence in detecting CLR. The system was further integrated into pruning solutions for Arabica coffee farms. A pruning device was designed using Autodesk Fusion 360^®^ and fabricated for testing on a coffee plantation in Indonesia.

## 1. Introduction

One of the world’s most popular beverages is coffee. Coffee’s unique flavor and aroma have made people around the world fall in love with the beverage and even incorporate it into their daily routines. However, the effects of climate change pose a threat to coffee production. Since coffee plants are very sensitive to weather, even small changes in temperature and rainfall patterns can have a big impact on the quality, quantity, and growth of the crop. The coffee leaf rust (*Hemileia vastatrix*) is a plant pathogen that attacks the organs of a coffee plant, particularly the Arabica variety. This disease causes the leaves to drop, leading to barren plants, dying shoots on the branches, and eventually the death of the plant.

Without a host, this fungus acts as an obligatory parasite that spreads through lightweight spores that are easily carried by the wind. When a plant is attacked, the fungus spreads rapidly by spores, either through wind or rain splashes, making it easy for nearby plants to become infected [1].

**Figure 1:**
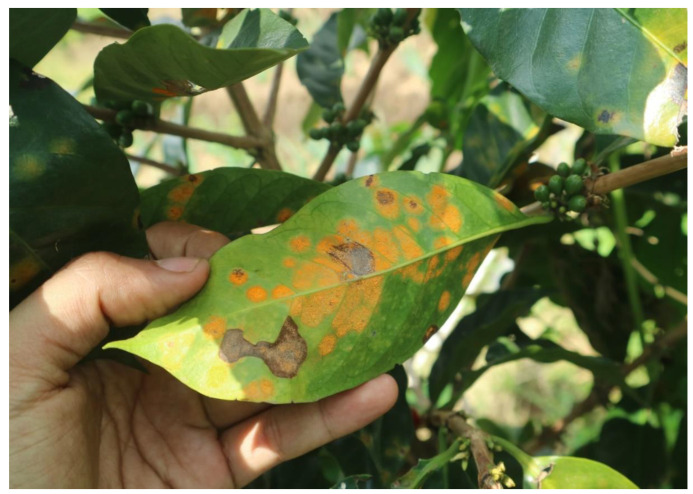
Coffee leaf rust disease under severe conditions.

The undersides of diseased plants’ leaves exhibit bright yellow spots that eventually turn dark yellow, then black, and dry out (Figure 1). An orange powder forms on the underside of the leaves. In extreme cases, the leaves fall off, gradually exposing the bare plant. Plant death occurs when starch reserves in the roots and branches of these plants are depleted. This disease is a major problem in Arabica coffee production [2]. Dark-colored structures, known as telia, form on infected leaves at this stage and release basidiospores. These spores can travel on the wind, facilitating the spread of disease. Wind is the primary medium for the spread of coffee leaf rust [1]. Because of their light weight, uredospore has a long range in the air. Similarly, wind currents carry basidiospores, if present. Coffee leaf rust can also be spread by human activities, including the use of contaminated equipment and the movement of diseased plant material. Although not the primary vectors, animals and insects can mechanically carry rust spores on their bodies.

Currently, pesticide spraying is known to be the most effective way to control coffee leaf rust disease [3]. This pesticide should be sprayed after the harvest period of coffee plantation. To increase the effectiveness of pesticide spraying, pre-spraying pesticide treatment, which involves pruning, should be carried out. This step is important for increasing the effectiveness of pesticide spraying. Pruning helps increase coverage, and selective or targeted pruning can potentially increase the effectiveness of pesticide spraying for managing coffee leaf rust (CLR) by creating a more targeted and accessible treatment environment [4]. 

Determining which leaves need to prune is important. In this case, pruning should only be performed on the most infected leaves and does not cut leaves that are still at an early stage of the disease. However, most farmers tend to cut all leaves affected by rust, even in the early stage, which can reduce coffee bean productivity due to lack of leaf density [5]. The use of image processing in computer vision offers a promising solution to identify which leaves need to be cut during the pruning process for managing coffee leaf rust disease. By developing a detection model using deep learning algorithms, researchers can analyze images from an originally collected dataset, allowing the model to accurately identify and distinguish between healthy and infected leaves. This approach not only improves the accuracy of the pruning process but also contributes to more effective disease management, ultimately supporting the health and yield of coffee crops.

This pattern can be effectively recognized by a deep learning model. Early disease detection is made possible by the model’s ability to predict whether a plant is affected based on a given dataset. In addition, delaying the onset of symptoms can lead to a reduction in the use of chemical pesticides [6,7]. Disease detection allows for the timely implementation of disease control measures to stop the spread of the disease. Therefore, to reduce production losses, a real-time detection model of coffee leaf rust is proposed, utilizing deep learning algorithms to support pruning decisions. Model detection can be used to detect CLR in real time; to make this possible, a mobile computing unit is needed to perform real-time detection in the field. Hence, the use of an edge device has also been proposed in this research. In this study, an edge device was used to deploy real-time detection of CLR to support pruning decisions. Therefore, this research aims to develop an automated detection method for coffee leaf rust for efficient pruning to mitigate the disease in coffee production.

## 2. Related Work

The goal of the artificial intelligence field of “deep learning” is to create computer models and algorithms that can understand and learn from data on their own. Artificial neural networks, the foundation of deep learning, are capable of ingesting, analyzing, and retrieving information from extremely complicated data [8]. Today, deep learning is known for its benefits in many domains, such as speech recognition, automatic spelling, and even facial identification. Plant disease identification is an interesting use case for deep learning. The traditional method of detecting plant diseases involves direct human visual inspection, which can be inaccurate, time-consuming, and inconvenient. Therefore, deep learning has made it possible to teach computer systems to more accurately identify and diagnose plant diseases.

Early identification or even simple detection of coffee leaf rust is the first step towards effective crop protection that can provide an opportunity to control rust disease using modern technology [7,9]. A study by [7] showed how modern technologies can be used for early detection of coffee leaf rust. The purpose of this study was to determine whether multispectral photos taken with an unmanned aerial vehicle (UAV) could accurately detect coffee leaf rust. The results showed an 80% detection accuracy.

To prevent coffee rust disease, most coffee-producing nations still employ conventional management techniques. Conventional CLR control methods rely heavily on fungicides and manual labor. Although these techniques provide some alleviation, they have disadvantages in terms of cost, environmental effects, and efficacy. To reduce the possibility of pesticide residues existing on coffee beans, pesticides are usually sprayed immediately after the harvest period. Prior to pesticide treatment, pre-spraying should be performed, and pruning of the most infected leaves can regulate the spread of the disease to other leaves.

Pruning helps increase coverage, and selective or targeted pruning can potentially increase the effectiveness of pesticide spraying for managing CLR by creating a more targeted and accessible treatment environment [4]. Selecting which leaves to prune is also crucial. In this instance, only the leaves with the highest level of infection are pruned, and leaves with an early stage of infection are left uncut. However, many farmers tend to eradicate leaf rust completely, even when it is still in its early stages, which may result in a decrease in coffee bean production. Farmers can quickly and accurately determine which leaves need to be pruned by using deep learning-based detection models to detect coffee leaf rust as a pruning option. By automating the process and minimizing human error, this method saves a significant amount of time compared to traditional visual detection by eye. As a result, the entire process becomes more efficient and effective, allowing growers to minimize effort, maximize resource utilization, and ultimately reduce production losses caused by improper or delayed pruning.

Owing to the separate nature of studies on CLR detection and pruning management, a gap exists that needs bridging to enhance integrated disease management. This study aims to address this gap by using deep learning algorithms to support decision-making in the pruning of CLR-infected leaves. By implementing advanced image recognition technologies, farmers can easily identify which leaves are most affected and should be pruned, thus minimizing errors in the pruning process. This approach not only streamlines the pruning decision but also ensures more precise and efficient disease control, ultimately leading to better crop health and yield.

## 3. Materials and Methods

### 3.1. Research Framework

**Figure 2:**
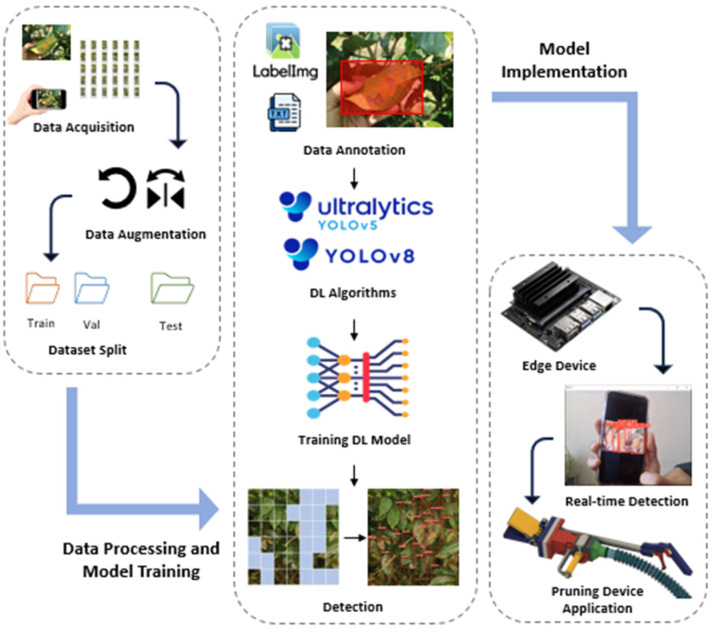
Conceptual research framework.

Data collection, data augmentation, data annotation, training, testing, and validation are critical steps in the process of creating an object detection model (Figure 2). First, data collection involves gathering images or video frames that contain objects of interest. These data should be diverse and representative of the different contexts in which the objects may appear. Data annotation is the process of labeling the collected data by marking the location and size of the objects within each image using bounding boxes. Annotation tools can facilitate this task and ensure consistency in labeling. Data augmentation is a technique used to increase the diversity of the training data and improve the model’s ability to generalize [10]. This process involves applying transformations such as rotation, flipping, and scaling to the images, creating variations that the model can learn from. By exposing the model to different perspectives and scenarios, data augmentation helps to prevent overfitting and improves robustness. Once the data are collected, annotated, and augmented, it is important to split the data into separate sets for training, validation, and testing. The training set is used to train the model, allowing it to learn the patterns and features associated with the objects. The validation set is used during the training process to tune hyperparameters and monitor the model’s performance to avoid overfitting and ensure that the model generalizes well to new data. The test set is reserved for evaluating the final performance of the trained model. It provides an unbiased assessment of the model’s accuracy and effectiveness in detecting objects, allowing the calculation of key metrics such as precision, recall, and mean average precision (mAP). This evaluation helps to understand how well the model performs in real-world scenarios and guides any necessary improvements or adjustments. The best-trained model will be deployed for real-time detection applications in the edge device as a model application and integrated with the pruning device in future work.

#### 3.1.1. Data Collection

**Figure 3:**
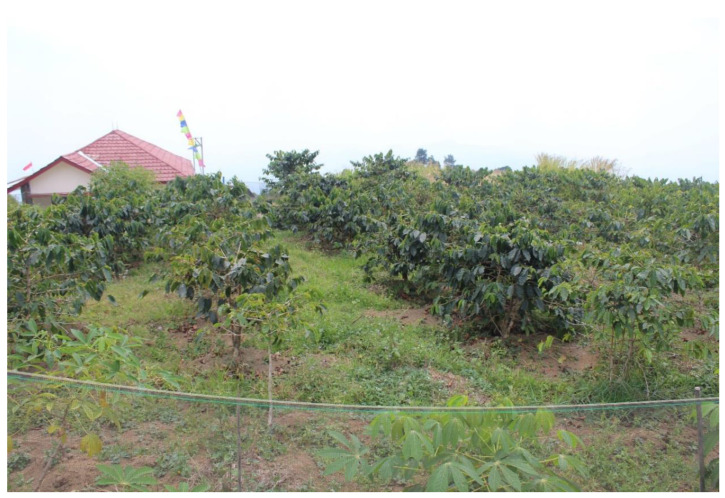
Arabica coffee farm in Majalengka District.

Data were collected from an Arabica coffee farm in Majalengka District, West Java Province, Indonesia (Figure 3). Majalengka is one of the 18 districts located in West Java Province, covering an area of 1204 km^2^. From a 2 ha farm, 20 sample trees were randomly selected to collect the dataset.

When building an object detection model, it is essential to consider the diversity and variability of the data it will be trained on [11]. A diverse dataset that includes images from different devices, angles, and methods can be advantageous for model performance. It exposes the model to a wide range of scenarios, lighting conditions, resolutions, and perspectives, which can help to learn to detect objects more robustly in different settings. Hence, in this study, the data were collected for object detection using two devices, a digital mirrorless camera, Canon EOS M3 (Canon Inc., Tokyo, Japan) and a phone camera, Redmi Note 10 (Xiaomi Corporation, Beijing, China). A total of 1025 images were collected after the videos were converted to images to create the dataset, and image cleaning was performed to eliminate blurry and unclear images, which were later used for training in model detection.

#### 3.1.2. Data Preparation

**Figure 4:**
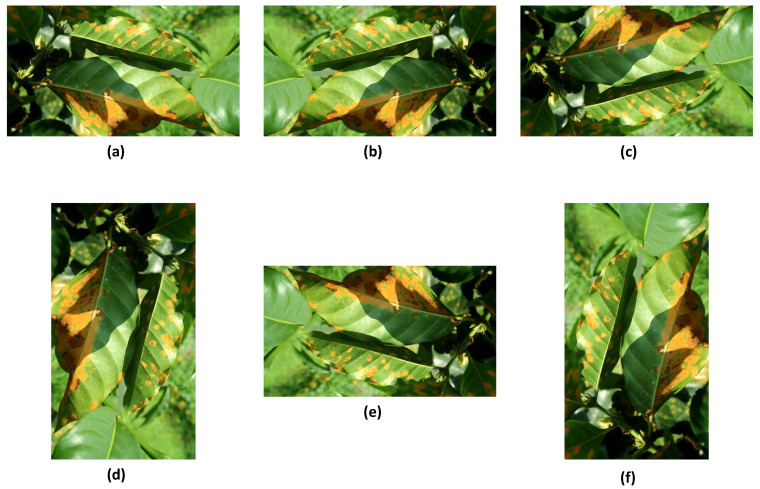
Image augmentation: (**a**) original image, (**b**) flipped horizontally, (**c**) flipped vertically, (**d**) rotated 90°, (**e**) rotated 180°, and (**f**) rotated 270°.

Data preparation was performed before the annotation process. Data augmentation was performed to increase the dataset size. Augmentation processes, such as rotation, flipping, and slicing, were applied to the images to diversify the dataset. A total of 2025 images were collected after the augmentation process (Figure 4). The dataset was divided into three sets of images, with a ratio of 70% for training (1417 images), 20% for validation (405 images), and 10% for testing (202 images).

#### 3.1.3. Data Annotation

Data annotation was conducted using the LabelImg tool, with data labeled as “CLR”. In this study, only the leaves in the most infected stage were labeled, as they are directly associated with pruning. This is accomplished by labeling the entire leaf rather than just the rust spots.

**Figure 5:**
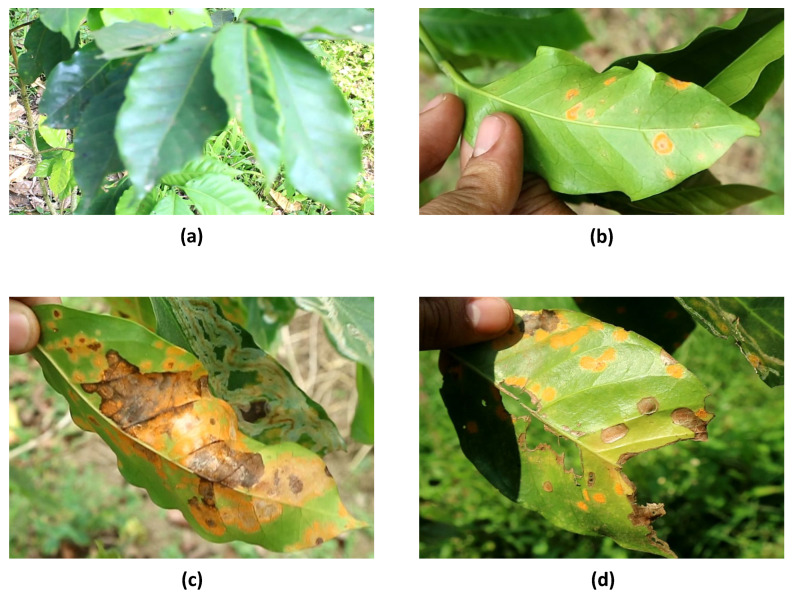
Stages of coffee leaf rust disease in the dataset: (**a**) healthy, (**b**) early stage, (**c**) severe stage with chlorosis, (**d**) severe stage with chlorosis and defoliation.

The first symptoms of CLR are yellow spots on the abaxial side of leaves [12]. These severe conditions are characterized by chlorosis and defoliation, which reduces its photosynthetic capacity [12]. Figure 5 shows that the collected dataset consisted of several coffee leaves in different stages of CLR. In this study, the images were divided into four stages: healthy, early stage, severe stage with chlorosis, and severe stage with chlorosis and defoliation. Thus, to provide labeling consistency, only the leaves with the most infected stages, which are the severe stage with chlorosis and defoliation, were manually labeled using a bounding box to safeguard the neural network detection performance. The labeled image generated a corresponding .txt file. These files contained information about the object class and the coordinates of the bounding boxes, representing the corners of the bounding boxes from the upper left to the lower right.

The labeled images were trained via the you only look once (YOLO) deep learning algorithm, which is a widely used algorithm in computer vision for object detection. Two YOLO versions were used, YOLOv5 and YOLOv8, for performance comparison.

#### 3.1.4. Data Training and Validation

YOLO is a one-stage object detection technique that has been in use since 2016. As one of the quickest object detection models, created by Joseph Redmon, YOLO can handle more than 45 frames per second on a GPU. This algorithm is known for its fast computation time and simple architecture, which directly provides the location and class of bounding boxes in a neural network [13]. YOLO divides the image into S × S grids to detect CLR infections [14]. The model predicts multiple bounding boxes and confidence scores for each box if the CLR disease center is within a given grid cell. However, since YOLO predicts many bounding boxes for each grid, non-maximum suppression (NMS) is used to find and remove unnecessary bounding boxes to accurately detect CLR disease at the end.

The annotated dataset was trained with two object detection algorithms, YOLOv5 and YOLOv8, for comparison and for utilizing the best model outcome for object detection. Because of their similar architectures, YOLOv5 and YOLOv8 are ideal for this research, providing effective, high-performance object recognition with a favorable trade-off between accuracy and speed, specifically for real-time detection. While their similar architecture allows for insightful comparisons, YOLOv8 offers sophisticated modifications that can improve recognition results, making it a useful standard for increasing accuracy in various applications.

**Figure 6:**
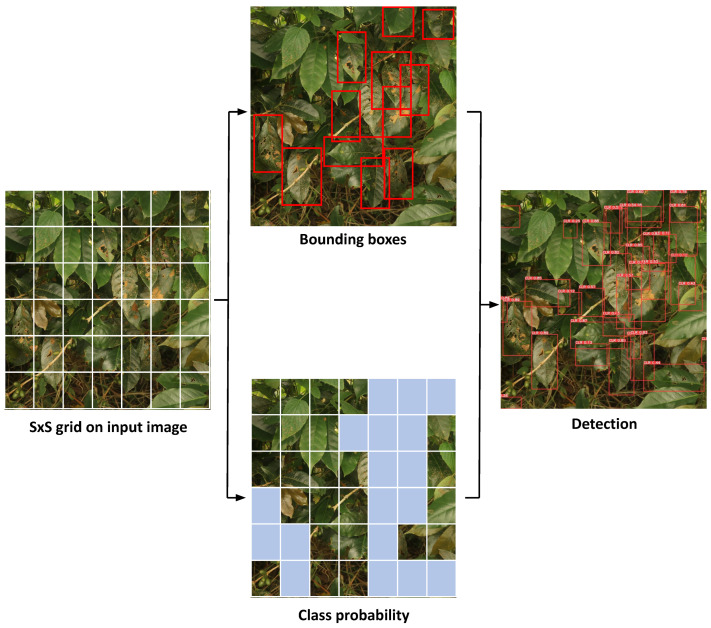
CLR detection process on the YOLO framework.

The annotated dataset includes bounding boxes that indicate the class probability of detection (Figure 6). The training process was conducted via two algorithms, YOLOv5 and YOLOv8. Standard evaluation metrics for object detection, such as intersection over union (IoU) and average precision (AP), were used to assess the model’s effectiveness in detecting CLR disease. These metrics are widely accepted for measuring the accuracy and performance of detection models.

The intersection over union (IoU) is a common metric for evaluating how accurately an object detection model localizes objects and measures any localization errors. It calculates the overlap between the predicted and actual bounding boxes. This involves determining the area where the predicted and ground truth bounding boxes intersect (the intersection) and the total area covered by both bounding boxes (the union). The IoU ratio was obtained by dividing the intersection by the union, which indicates how closely the predicted bounding box matches the actual bounding box.

Precision (P), on the other hand, measures the model’s accuracy in detecting CLR by calculating the proportion of true positives out of the total predictions made by the model [15]. It can be computed via the following equation:

(1) $$\mathrm{Precision}=\frac{\mathrm{TP}}{\mathrm{TP}+\mathrm{FP}}.$$

Recall is a metric that evaluates the model’s effectiveness in accurately identifying all instances of CLR, including those that were initially overlooked [16]. It is calculated as the ratio between the number of correct predictions made by the model for a specific class and the total number of actual instances of that class present in the dataset. This metric helps determine how well the model captures all positive targets, providing insight into its thoroughness in detecting the disease.

(2) $$\mathrm{Recall}=\frac{\mathrm{TP}}{\mathrm{TP}+\mathrm{FN}},$$

where TP is the true positive value (i.e., the correct detection box); FP is the false positive value (i.e., the false detection box that predicts the background as the target); and FN is the false negative value (i.e., the missed detection box).

A trade-off between recall and precision may be shown by varying the categorization threshold, which results in a curve. The average precision for each class in the model is represented by the area under this precision‒recall curve. The mean average precision (mAP) may be computed by taking the average of these values over all classes, which can be performed via the following formula:

(3) $$\mathrm{mAP}=\frac{1}{C}\sum_{k=1}^{T}P(k)\text{∆}R(k)$$

where C is the total class number, T is the IoU threshold number, k is the IoU threshold, P(k) is the precision, and R(k) is the recall.

#### 3.1.5. Edge Device Implementation

The YOLO model with the best performance was selected for real-time application on an edge device. The model was deployed on a single-board computer, the NVIDIA Jetson Nano, integrated with a camera. The Jetson Nano was equipped with a 4GB GPU, which provides notable inference speed and supports the computing requirements of deep neural networks. Real-time performance was tested to detect CLR-infected leaves, with input images streamed directly from the camera to the YOLO model running on the Jetson Nano. The images of healthy leaves were also streamed to verify the model’s ability to avoid misdetections. This system was then mounted on a pruning device to handle detection tasks in real-world applications. A Python script can further automate the cutting actuator, enabling the system to act upon detecting leaf rust.

## 4. Results

### 4.1. Training Configurations

The training process was performed for each detection model with the same number of training parameters, including epochs, image size, and batch size (Table 1).

Setting the same parameters, such as epochs, batch size, and image size, during YOLOv8 and YOLOv5 training is essential for ensuring consistency and stability in the training process. First, maintaining consistent epochs allows the model to undergo the same number of iterations or training cycles, ensuring that it has sufficient opportunities to learn from the dataset and converge toward optimal performance. Deviating from consistent epoch numbers might result in incomplete learning or overfitting if the model is trained for too long or underfitting if it is trained for too short of a duration. Second, keeping the batch size constant ensures that the model processes the same number of samples in each iteration during training. Consistency in batch size helps stabilize the learning process, improves gradient estimation accuracy, and facilitates smoother convergence of the training process. Deviating from consistent batch sizes could lead to training instability, slower convergence, or degraded performance. Finally, maintaining consistent image sizes across training samples ensures uniformity in the input data fed into the model during training. The consistency of image sizes enables efficient processing and memory utilization, as well as consistent feature extraction across different samples. Deviating from consistent image sizes might introduce inconsistencies in the learned features, leading to suboptimal performance or training instability.

In essence, setting the same parameters for epochs, batch size, and image size in YOLOv8 and YOLOv5 training ensures stability, consistency, and efficiency throughout the training process, ultimately facilitating better convergence and improved model performance.

**Figure 7:**
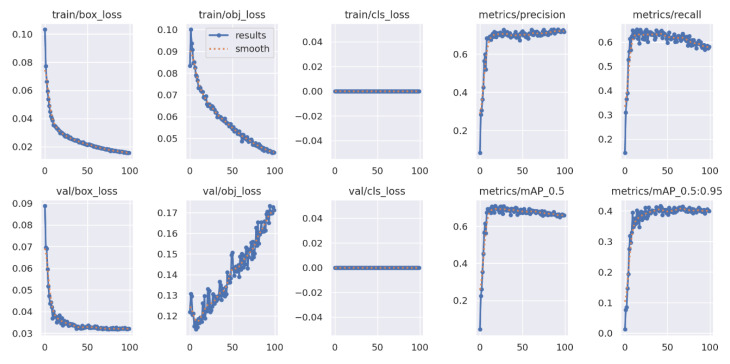
YOLOv5 training configuration.

All labeled images were trained with the YOLOv5 algorithm through the CNN and resulted in the training configuration shown in Figure 7. The training process with the pretrained yolov5m.pt model took 5 h and 10 min with 100 epochs; the image size was 416, and the batch size was 16. After the trained model was tested, CLR was detected by the machine in each frame. The trained model had a mean average precision (mAP) of 69.9%, and the recall was 63.4%. The mean average precision was low because of the small number of epochs. The dataset was trained with 100 epochs. The mean average precision, as shown in Figure 7, indicates that the model stopped learning after 80 repetitions (epochs). Thus, further training and a greater number of epochs are recommended for better detection. The matrix describes the results, and several parameters are used, such as precision, loss, and mean average precision (mAP).

**Figure 8:**
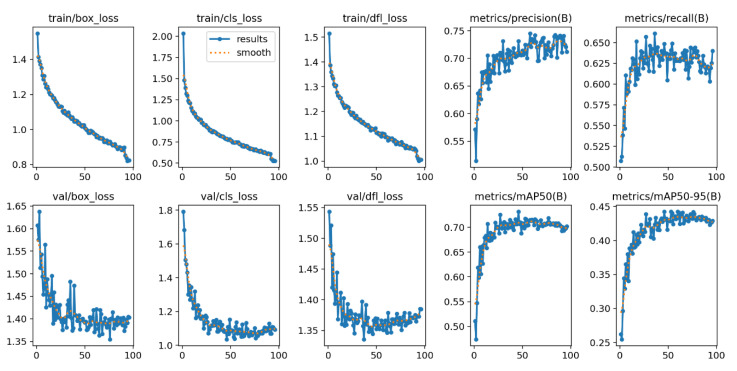
YOLOv8 training configuration.

The labeled images were also trained with the YOLOv8 algorithm through the CNN and resulted in the training configuration shown in Figure 8. The training process with the pretrained yolov8m.pt model took 6 h and 10 min with 100 epochs; the image size was 416, and the batch size was 16. After the trained model was tested, CLR was detected by the machine in each frame. The trained model had a mean average precision (mAP) of approximately 70.2% and a recall of approximately 64%. The mean average precision was significantly lower than expected because of the smaller number of epochs and the small dataset. The dataset was trained with only 100 epochs. Table 2 shows the comparison between the results of the YOLOv5m and YOLOv8m training configurations.

### 4.2. Model Testing

CLR was detected with some variety in precision. This was due to several factors that might influence the detection results, such as human error in the data annotation process. In such cases, the model may be biased toward the class due to the variety of objects in the bounding boxes during the annotation process, resulting in low precision. The model may struggle to correctly identify instances of CLR, leading to a greater number of false positives. Insufficient training data and deep learning models require a diverse and representative training dataset to learn the patterns and features of the target class accurately. If the dataset used for training is small or lacks variability, the model may not generalize well to unseen data, leading to low precision in detection. Increasing the size of the training dataset or using data augmentation techniques can help mitigate this issue. Complex or ambiguous class boundaries, such as distinguishing between various stages or severities of CLR, can have complex or ambiguous class boundaries [17]. Deep learning models may struggle to classify such instances accurately, resulting in lower precision. In such cases, it may be necessary to refine the dataset, provide clearer labels or annotations, or explore alternative approaches such as multiclass classification or object detection. The choice of model architecture and hyperparameters can greatly impact precision. If the model architecture is not suitable for the detection task or is not capable of capturing the relevant features, it may result in low precision. Additionally, hyperparameters such as the learning rate, regularization techniques, or optimizer selection can influence the model’s ability to generalize and discriminate between classes. Experimenting with different architectures and hyperparameter settings may help improve precision.

To evaluate the performance of the two trained models in detecting CLR, 202 original images, consisting of images from a digital mirrorless camera and a phone camera, were used for testing with the best-trained weight from each model. To further evaluate the robustness and generalizability of the model, it was then tested with a different dataset captured using 100 images from a digital mirrorless camera and 100 images from a phone camera. The images from the digital mirrorless camera and phone camera in the testing dataset were separated to test and assess the performance of the model in detecting different image qualities.

The results shown in Table 3 indicate that YOLOv8 was the most accurate in detecting CLR compared with YOLOv5. On the testing dataset, YOLOv8 achieved a higher mAP of 73.2%, with YOLOv5 scoring a slightly lower mAP of 69.9%. Additionally, YOLOv8 demonstrated better testing performance than YOLOv5 when it was evaluated on the dataset including images from the digital mirrorless camera, particularly in terms of precision, recall, and mean average precision (mAP). YOLOv8 had a mAP of 71.6%, whereas YOLOv5 had a mAP of 66.9%. Moreover, on the phone camera dataset, compared with YOLOv5, which had a mAP of 66.7%, YOLOv8 again had better testing performance, with a mAP of 71.9%. With these comparative evaluations, YOLOv8 demonstrated better performance than its predecessor, YOLOv5, specifically in the task of detecting CLR via a specialized test dataset.

One of the key metrics used to evaluate object detection models is the mean average precision (mAP), which balances precision (the accuracy of the positive predictions) and recall (the ability of the model to find all relevant instances).

**Figure 9:**
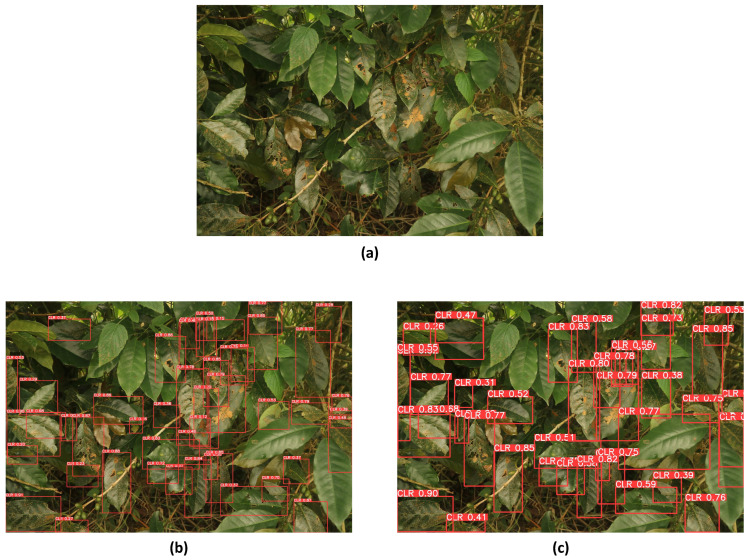
Detection results on the testing dataset comprising images from the digital mirrorless camera: (**a**) original image, (**b**) YOLOv5 detection, and (**c**) YOLOv8 detection.

The higher resolution and richer detail captured by digital mirrorless cameras provided a challenging testing environment, yet YOLOv8 excels in this context. The precision, which measures the accuracy of the positive detections, was significantly higher in YOLOv8, indicating fewer false positives. Recall, which assesses the model’s ability to identify all relevant instances, was also improved, showcasing YOLOv8’s enhanced capability to detect even the subtle signs of CLR. The detection results from YOLOv8 demonstrated a marked improvement over YOLOv5 (Figure 9), particularly in detecting instances of CLR. YOLOv8’s advanced architecture and enhanced object detection capabilities enable it to identify and localize a greater number of instances with higher accuracy. This is evident from the precision of the bounding boxes generated by YOLOv8, which exhibit various precision levels, accommodating the complexity and variability of the rust spots on the coffee leaves.

**Figure 10:**
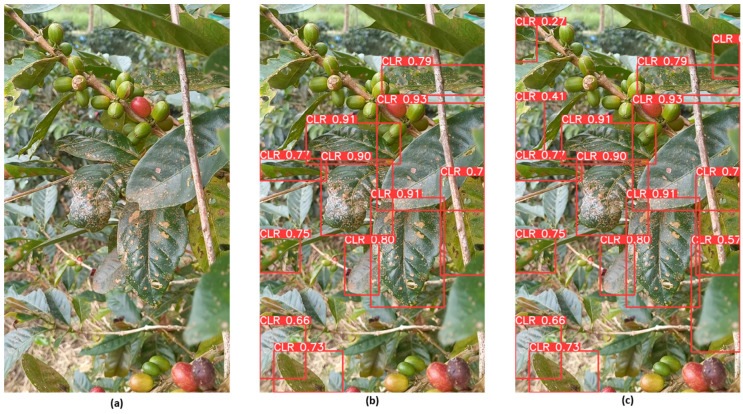
Detection results on the testing dataset comprising images from the phone camera: (**a**) original image, (**b**) YOLOv5 detection, and (**c**) YOLOv8 detection.

Testing the model with a phone camera dataset introduces a variety of image qualities, lighting conditions, and angles, presenting a realistic and diverse set of challenges. By subjecting the model to this new dataset, researchers can thoroughly assess its precision, recall, and mean average precision (mAP) in different scenarios. The insights gained from this phase of testing were invaluable for understanding the model’s practical applicability for real-time, on-the-go agricultural monitoring and disease detection. Ensuring that the model performs well with phone camera images was demonstrated for deployment in diverse and dynamic field conditions, where farmers and agricultural workers can use their smartphones to monitor crop health. In the testing conducted on the dataset consisting of images captured with a phone camera, YOLOv8 outperformed YOLOv5 in terms of detection accuracy (Figure 10). This superiority stems from YOLOv8’s ability to detect a greater number of objects with varied precision levels in the generated bounding boxes. The advanced architecture and refined algorithms of YOLOv8 enabled it to handle the diverse image qualities and lighting conditions typical of phone camera images more effectively.

By providing more accurate and reliable detection, YOLOv8 aids in the timely intervention and management of CLR, ultimately helping to protect coffee yields and support the livelihoods of coffee farmers. This comparison highlights the significant advancements in deep learning-based object detection models and highlights how continuous innovation in this field can lead to practical solutions for pressing agricultural challenges.

### 4.3. Real-Time Detection Using an Edge Device

YOLOv8 demonstrated better performance than YOLOv5 in detecting CLR, highlighting its capability for real-time detection systems in precision agriculture. This advancement offers potential for proactive disease management and crop health monitoring [18]. However, the practical deployment of real-time systems faces challenges due to the limitations of the Jetson Nano. While Jetson Nano supports deep learning models such as YOLOv8, its limited processing power and memory capacity hinder consistent real-time performance. Specifically, detection was performed at 10 fps (frames per second), resulting in reduced responsiveness and delay in identifying CLR, compromising the system’s efficiency.

**Figure 11:**
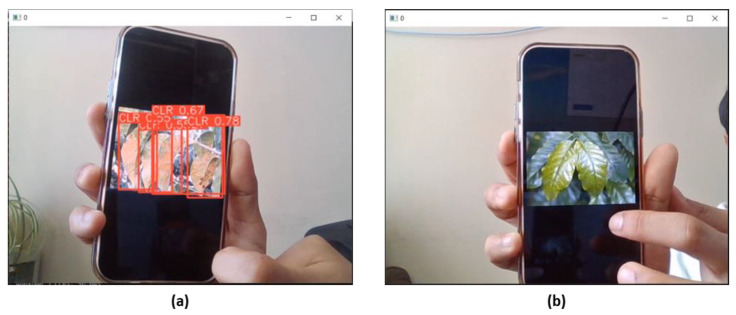
Real-time detection of CLR on Jetson Nano using YOLOv8: (**a**) infected and (**b**) healthy images.

Real-time detection was tested on two sets of testing images: infected leaves and healthy leaves (Figure 11). The images were provided by the coffee farmers from Majalengka District, Indonesia. The farmers selected images of the leaves they suspected to be diseased, as there was no access to nearby coffee farms during the testing period in Japan. Their contribution provided valuable data and ensured the model addressed real challenges in the field. This collaboration highlighted the importance of involving farmers in developing practical solutions for disease detection and management.

## 5. Discussions

### 5.1. Evaluation of the Dataset Distribution and Deep Learning Algorithms

Owing to the lack of publicly available CLR datasets for reference in data acquisition, an original CLR dataset was collected as part of the experiments using two different camera devices. The data collection process was challenging from the start, largely due to environmental factors that affected image quality and consistency, such as erratic weather and uneven lighting. Access to the coffee farms was made even more difficult and time-consuming by the rural road conditions in the Majalengka District. The diversification of the dataset, which included images captured by both mirrorless digital cameras and cell phone cameras, was intended to increase the model’s flexibility in detecting CLR under different conditions. This approach ensures that the real-time detection application can perform reliably under different conditions, whether in controlled environments or directly in the field, making the model more robust and adaptable for practical use. The dataset comprising images from the phone camera was intended to represent the use of lower-resolution cameras implemented in the edge device for the integrated pruning system. Since edge devices often have limitations in processing power and storage capacity, running high-resolution cameras may not be feasible. By training the model on images captured by phone cameras, the system works effectively within these constraints, ensuring that the integrated pruning device can perform accurate detection and pruning via the more accessible, lower-resolution cameras typical of edge devices.

**Figure 12:**
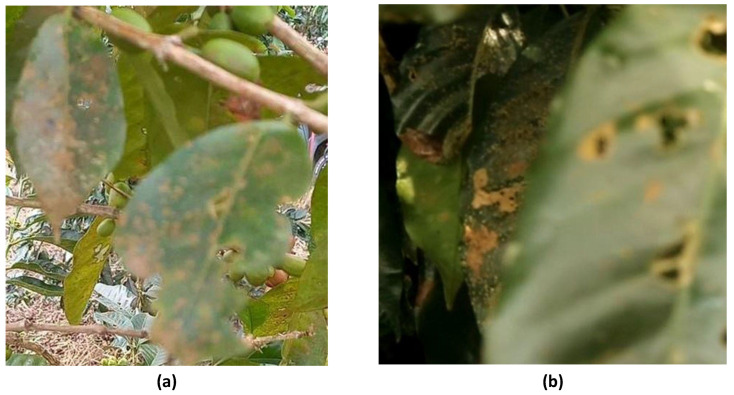
Challenge in dataset quality: (**a**) low-quality image (blurry) and (**b**) occluded object.

The performance of the model is highly dependent on the quality of the collected dataset, as the dataset serves as the basis for the model’s learning and recognition capabilities. In this research, certain images within the dataset were found to be blurry or of lower quality (Figure 12), which could influence the model’s ability to accurately detect and differentiate between healthy and diseased leaves. Low-quality images can obscure the fine details needed to identify the rust spots characteristic of coffee leaf rust disease [19], leading to potential misclassifications.

Another significant challenge in the dataset was leaf occlusion, where the target leaf infected by coffee leaf rust is partially occluded by other objects, such as branches, stems, or overlapping leaves (Figure 12). This occlusion makes it difficult to properly annotate rust-infected areas, and can sometimes cause confusion or inconsistencies during the annotation process [20]. These limitations not only affect the quality of the annotations but also influence how the model learns to detect coffee leaf rust, potentially reducing its accuracy in applications. Addressing these issues by refining the dataset or using advanced techniques to handle occluded objects was critical for improving the robustness and performance of the detection model.

The deep learning algorithms YOLOv8 and YOLOv5 were employed for detecting and classifying instances of CLR by analyzing input images. In this research, the YOLOv5m and YOLOv8m models were employed with a focus on achieving high detection accuracy, as the primary goal was to accurately identify leaves to be pruned during the cutting process. Both models struck an optimal balance between performance and computational efficiency, making them well suited for use on devices with low to moderate GPU power, such as edge devices, without sacrificing detection accuracy. By using these models, the study ensured that accurate detection was maintained while still being feasible for real-time applications in resource-constrained environments. YOLOv8 achieved higher precision, meaning that it generates fewer false positives, where healthy leaves are incorrectly identified as rust-affected. It also boosts improved recall rates, successfully identifying a greater number of true positive instances of CLR, thereby reducing the incidence of false negatives, where affected leaves were missed. These improvements increase the overall accuracy of detection [21]. The model’s limitations are significantly influenced by the constraints on data availability and the relatively small size of the dataset used for training and evaluation, which highlights the need for more extensive and varied data collection efforts. By incorporating a wider range of conditions and scenarios into the training dataset, the model can develop a more robust understanding of the task, leading to improved generalizability and performance in real-world applications.

### 5.2. Performance Analysis of the Edge Device for Real-Time Detection

Real-time detection using the edge device was successfully implemented, with the model effectively distinguishing between infected and healthy leaves. However, a performance limitation was identified in the Jetson Nano during real-time detection. Due to its lower processing power compared to standard computers, the detection performed at reduced frame rates, achieving only 10 fps. This performance is due not only to the device’s hardware limitation but also to the model’s size, which likely exceeded the processing capabilities of the edge device. Future work should focus on developing a more lightweight deep learning model to enhance real-time performance on the Jetson Nano [22].

Addressing these limitations is crucial to ensuring the scalability and effectiveness of real-time detection solutions in agricultural applications. Mitigating these technical challenges will enable edge device-based real-time detection systems to achieve their full potential in transforming disease management practices, including the integration of a CLR detection model with a pruning device as part of integrated disease management.

### 5.3. Deployment of the CLR Detection Model on the Pruning Device

For implementation, the pruning support device was designed as a deployment medium for the CLR detection model. This device was meticulously designed using Autodesk^®^ Fusion™ software, leveraging its advanced modeling, simulation, and rendering capabilities to create a highly functional and efficient tool. The design was tailored for practical use by farmers in pruning operations. The following section discusses the design parameters for CLR detection and the proposed pruning solution.

**Figure 13:**
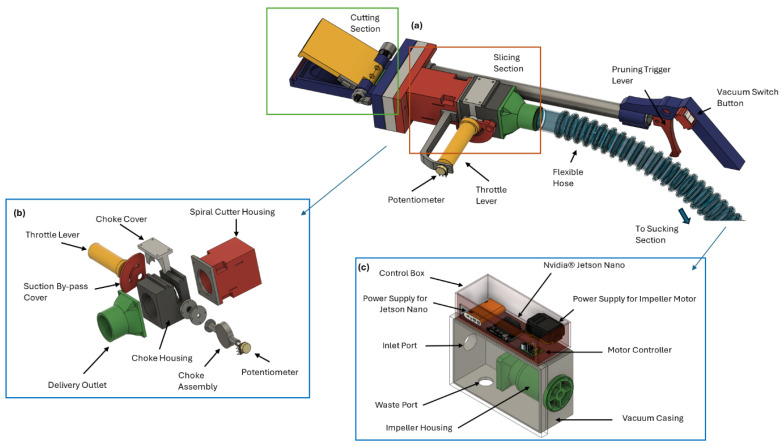
Design of the pruning device components. (**a**) Pruning device, i.e., cutting part, (**b**) slicing part, and (**c**) vacuum tank.

The cutting part of this device was divided into two important mechanisms (Figure 13): the cutting section and the slicing section. The cutting section was specifically equipped with a suction cup with a cutting blade, which can be easily controlled with a pruning trigger lever, and a design was proposed to adjust the size of the coffee leaf from small to large. The slicing section chops the leaf that was cut to make the suction process easier. The pruning device was integrated with a camera as an object recognition tool that connected to the Jetson Nano edge device as the main computing unit, enabling it to scan each coffee leaf for signs of rust. The camera captured detailed footage of the leaves, which were then analyzed in real time via an embedded algorithm that employs machine learning techniques to identify rust infections accurately. This precise detection capability allows the device to distinguish between healthy and infected leaves with a high degree of accuracy.

The pruning device was connected to a vacuum tank through a flexible hose, which was designed to suck the chopped leaf into the vacuum tank to ensure that the leaf that needs to be cut does not fall to the ground, since there is still a possibility for infection through wind transfer. The power voltage of the vacuum was designed to be set with a potentiometer integrated with a throttle lever. The cutter was designed to trim the identified leaf precisely, ensuring minimal damage to the surrounding healthy foliage. Once cut, the leaf was directed into a suction cup, which swiftly pulled it into a connected vacuum system. This vacuum transported the pruned leaf into a secure residue box through a flexible hose, ensuring that the infected material was safely contained and removed from the plantation. This automated process not only increases the efficiency of managing CLR but also reduces the labor and time required for manual pruning.

Before the pruned leaf is drawn into the vacuum system, the leaf passes through a slicing mechanism integrated with radial blades inside the spiral cutter housing (Figure 13). The radial blades are designed to finely chop the leaf into smaller pieces, making it easier for the vacuum to efficiently suck up the cut fragments. The slicing mechanism was designed to ensure that the leaf was reduced to a manageable size, preventing any blockage and enhancing the overall efficiency of the vacuum system. This additional step not only facilitates smoother operation but also ensures that the infected leaf material is thoroughly processed before being securely stored in the vacuum box, contributing to more effective and streamlined management of CLR.

The vacuum tank was designed to be attached to the control box. The control box contained the Jetson Nano device as the main computing unit for the real-time detection of CLR and a power bank to supply power. A power supply battery is also connected to the motor controller to adjust the vacuum power for suction performance. Once a rust-infected leaf is pruned by the device, it is immediately sucked into an integrated vacuum system (Figure 13). This vacuum, connected through a flexible hose, ensures the swift and clean removal of the pruned leaf from the plant. The pruned leaf passes through the flexible hose and is deposited into a designated residue compartment within the device’s power box. This compartment was designed to securely store the collected leaves, preventing any potential spread of the disease back into the environment. The seamless operation of pruning and suction not only streamlines the removal process but also enhances the overall hygiene and efficiency of coffee plant maintenance, ensuring that rust-infected leaves are handled swiftly and contained properly. When the leaf is sucked into the vacuum through the intake port, it is swiftly transported into the vacuum box. Inside this compartment, the chopped leaf fragments, including the rust-infected material, are securely stored. This containment ensures that the rust spores are isolated and prevents them from spreading back into the environment. Once the vacuum box reaches its capacity or at designated intervals, the accumulated residue can be easily disposed of through the waste port. This port is designed for convenient and hygienic removal of leaf rust debris, allowing for quick disposal without direct contact with the infected material. This efficient waste management process ensures that the device operates continuously and maintains a clean and effective pruning system.

**Figure 14:**
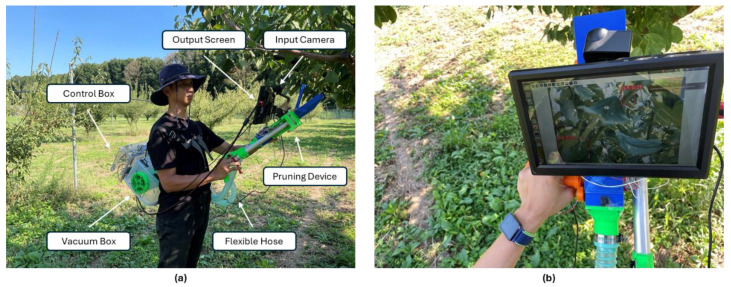
Design of the test for real-time detection before deployment in the field at an Arabica coffee farm: (**a**) Device components and (**b**) real-time detection.

The CLR pruning support device was fabricated with the successful 3D printing of several essential components. These parts were designed and printed to ensure precision and functionality. Following the assembly, the detection model was deployed using the NVIDIA Jetson Nano edge device. This edge device was connected to a camera that serves as a recognition tool for identifying rust-affected coffee leaves. The integration of the Jetson Nano device with the camera enabled real-time detection and facilitated the efficient pruning of infected leaves, enhancing the overall effectiveness of the device. Figure 14 shows the test design of the pruning support device to show how it is used. Real-time testing of the pruning device will be carried out on a coffee farm in the future.

As another implementation of real-time CLR detection, using a mobile phone-based program to implement the recognition model as an alternative to real-time recognition is also possible. The built detection model is believed to be able to use platforms such as TensorFlow Lite, which can run on mobile devices, making it easy for farmers to recognize which leaves need to be pruned in real time. Farmers would be able to use this cutting-edge detection technology directly from their mobile phones, which would provide a very affordable and accessible tool. Incorporating this feature, further research needs to be carried out to improve the deployment of the device to the small and remote farms.

## 6. Conclusions

Coffee leaf rust (CLR; *Hemileia vastratrix*) is a disease that threatens coffee production. Pruning the leaf most infected with CLR is one of the important steps in disease management before pesticide spraying becomes more effective. To address this challenge of detecting CLR and facilitate real-time pruning decisions to stop the infection caused by rust disease, this research yielded the following outcomes.

### 6.1. Dataset Contributions

An original dataset of coffee leaf rust (CLR) was collected via two devices: a mobile phone camera and a digital mirrorless camera. This approach was designed to develop a model capable of flexibly detecting CLR in images of different qualities, ensuring robust performance in different scenarios. This combination allowed the model to perform real-time detection effectively regardless of the camera quality in actual field situations. The purpose of dataset diversification is to create model flexibility to detect CLR in the testing dataset under different conditions and in different environments for real-time applications. As YOLOv8 demonstrated better performance than YOLOv5 achieved in detecting coffee leaf rust, its capabilities were leveraged for the implementation of real-time detection systems, deployment in edge devices, and implementation in pruning devices. This dataset can be used for further implementation and collaboration related to coffee leaf rust disease management, especially in computer vision.

### 6.2. Model Development for CLR Detection

A model was developed for detecting coffee leaf rust using a deep learning algorithm to support pruning decisions. After the trained model was tested, coffee leaf rust was detected in each frame. The trained YOLOv5 model has a mean average precision (mAP) of 69% and a recall of 63.4%. Meanwhile, the trained YOLOv8 model has a mean average precision (mAP) of 70.2% and a recall of 65.9%. Compared with YOLOv5, YOLOv8 was the most accurate at detecting coffee leaf rust. On the testing dataset, YOLOv8, with a mAP of 73.2%, outperforms YOLOv5, with a mAP of 70.5%. The detection results from YOLOv8 demonstrated a remarkable improvement over YOLOv5 on a dataset comprising images from a digital mirrorless camera and a dataset comprising images from a phone camera.

### 6.3. Edge Device Implementation for Real-Time Detection of CLR for Pruning

The application of real-time detection via the Jetson Nano edge device was successfully carried out, and satisfied preliminary results were obtained for real-time detection. This system could detect CLR and provide detection output with sufficient accuracy, in accordance with the initial development goals. However, a significant shortcoming in its implementation was the delay in the output display, which affects the overall response speed of the detection system. This delay was caused mainly by the limited performance of the Jetson Nano edge device, which, although efficient and low in power, has limitations in terms of fast data processing capabilities. The use of the Jetson Nano edge device in this study for real-time detection and integration with a pruning device was one of the first attempts ever made. The Jetson Nano works as the system’s central processing unit, handling the data received from the camera and executing deep learning algorithms to facilitate optimal pruning decisions. This combination of technologies facilitates enhanced productivity and precision in the pruning process.

Further research will be carried out on the integration of the real-time detection of CLR and mechanical pruning devices to create integrated CLR disease management, and the device will be tested on a real Arabica coffee farm. The model performance can also be enhanced by collecting more diverse datasets under different environmental conditions. Real-time testing in the field will be performed to evaluate the performance of the pruning device, along with the performance of farmers and stakeholders, to obtain their views as experienced professionals in coffee cultivation.

## Figures and Tables

**Table 1:** Training parameters for the detection model.

Model	Input Size	Batch Size	Epoch
YOLOv5m	416 × 416	16	100
YOLOv8m	416 × 416	16	100

**Table 2:** Summary of the results of the YOLOv5m and YOLOv8m training configurations.

Model	Precision (%)	Recall (%)	mAP (%)
YOLOv5m	71.8	63.4	69.9
YOLOv8m	73.2	64.9	70.2

**Table 3:** Testing the performance of the deep learning algorithm in detecting CLR via a variety of testing datasets.

Model	Testing Dataset			Digital Mirrorless Camera Dataset			Phone Camera Dataset		
	Precision (%)	Recall (%)	mAP (%)	Precision (%)	Recall (%)	mAP (%)	Precision (%)	Recall (%)	mAP (%)
YOLOv5m	69.9	63.4	69.9	66.1	60.1	66.9	68.1	62.1	66.7
YOLOv8m	73.2	65.9	73.2	70.9	69.5	71.6	69.9	70.5	71.9

## Data Availability

The dataset that was generated and used in this study is available on request from the corresponding author, but restrictions apply to data reproducibility and commercially confidential details.
